# Multiparametric quantification of thermal heterogeneity within aqueous materials by water ^1^H NMR spectroscopy: Paradigms and algorithms

**DOI:** 10.1371/journal.pone.0178431

**Published:** 2017-05-26

**Authors:** Norbert W. Lutz, Monique Bernard

**Affiliations:** Centre de Résonance Magnétique Biologique et Médicale, Unité Mixte de Recherche 7339, Centre National de la Recherche Scientifique, Faculté de Médecine de la Timone, Aix-Marseille Université, Marseille, France; California State University Fresno, UNITED STATES

## Abstract

Processes involving heat generation and dissipation play an important role in the performance of numerous materials. The behavior of (semi-)aqueous materials such as hydrogels during production and application, but also properties of biological tissue in disease and therapy (e.g., hyperthermia) critically depend on heat regulation. However, currently available thermometry methods do not provide quantitative parameters characterizing the overall temperature distribution within a volume of soft matter. To this end, we present here a new paradigm enabling accurate, contactless quantification of thermal heterogeneity based on the line shape of a water proton nuclear magnetic resonance (^1^H NMR) spectrum. First, the ^1^H NMR resonance from water serving as a "temperature probe" is transformed into a temperature curve. Then, the digital points of this temperature profile are used to construct a histogram by way of specifically developed algorithms. We demonstrate that from this histogram, at least eight quantitative parameters describing the underlying statistical temperature distribution can be computed: weighted median, weighted mean, standard deviation, range, mode(s), kurtosis, skewness, and entropy. All mathematical transformations and calculations are performed using specifically programmed EXCEL spreadsheets. Our new paradigm is helpful in detailed investigations of thermal heterogeneity, including dynamic characteristics of heat exchange at sub-second temporal resolution.

## Introduction

Temperature control in production and application of aqueous materials such as hydrogels requires detailed insight into thermal properties, notably heat conduction and dissipation. Hydrogels were the first biomaterials developed for human use [1], and in recent years a wide range of functional, structural and dynamic properties of hydrogels, including also "intelligent" or "smart" hydrogels, have been studied by a variety of methods [2–11], including nuclear magnetic resonance (NMR) [4, 5, 12]. Further research has focused on temperature-dependent behavior of hydrogels [13, 14]. Moreover, investigation of temperature regulation in biological tissue has gained renewed interest in view of modern medical techniques such as hyperthermia, cryotherapy/cryosurgery, tissue cutting/welding with lasers, and transplantation of organs after frozen storage [15–19]. In all these cases, considerable temperature gradients occur within the materials in question, and change significantly over time. This highlights the necessity to not only measure average temperature values, but to quantify thermal heterogeneity within these (semi)-aqueous materials. Although temperature mapping for individual selected slices of body tissue has recently been used in clinical environments [19–22], this approach does not provide parameters that characterize thermal heterogeneity in a *quantitative* manner. By contrast, an adequate evaluation of the frequency of occurrence (= frequency distribution) of all temperature values present within a given sampled volume, at a given time point, would enable quantitative analysis of the characteristics of thermal heterogeneity. To address this challenge, we propose a new paradigm allowing, for the first time, examination of the statistical distribution of temperature values, resulting in at least eight different heterogeneity parameters for many water-containing materials. This is achieved experimentally by multiparametric analysis of the ^1^H NMR signal of water, an intrinsic "physicochemical temperature probe" for (semi-)aqueous materials.

Generally, the exact position (chemical shift) of a water ^1^H NMR signal varies with the sample temperature in a linear fashion (ca. 0.01 ppm/°C) [23–25], primarily as a consequence of temperature effects on the number, lengths and angles of hydrogen bonds [24, 26]. This relationship has been exploited to determine one single temperature value for a given sample volume (or volume element = voxel). The use of the water ^1^H NMR signal for temperature measurement in a hydrogel-loaded cell perfusion system has been demonstrated more than 20 years ago [27], and was further developed for in vivo applications based on selected cross sections of biological tissue [28–30]. A large number of studies were aimed at clinical applications [31–33]. Conventionally, the chemical shift of the highest point ("the" maximum) of the water ^1^H NMR resonance is converted to a temperature value based on a calibration curve, and this value is interpreted to indicate "the" temperature of the measured volume or volume element (voxel) [27, 30]. Although this procedure yields fairly realistic average temperature values for narrow and symmetric temperature distributions, it is inadequate when a temperature distribution deviates from this ideal shape due to significant thermal heterogeneity within the volume represented by the NMR spectrum. By contrast, our qualitatively new approach is based on the circumstance that a water ^1^H NMR signal obtained from aqueous material in which temperature gradients exist, represents the entire temperature distribution throughout the underlying volume, rather than merely a single, averaged temperature value. We convert the entire water resonance into a temperature curve, then exploit the shape of this temperature distribution profile via its histogram to derive the following quantitative parameters adapted from classical statistics: one or multiple temperature modes (= curve maxima); weighted mean and median temperatures [34], each of which takes into account the entire temperature distribution; temperature range; and asymmetry (skewness [35]), peakedness (kurtosis [34, 36]) and smoothness (entropy [37, 38]) of temperature distributions. Finally, ratios of areas under individual temperature modes and/or ranges are determined to obtain a quantitative measure of the relative sizes of volumes with characteristic temperature ranges. This method does not require imaging technology, and can therefore be implemented in widely available analytical NMR spectrometers. Moreover, it is sufficiently fast to follow changes in temperature profiles over time at a rate of multiple measurements per second. Statistical analysis of water ^1^H NMR line shapes as a method for contactless quantitative analysis of thermal heterogeneity in (semi-)aqueous materials may first and foremost find broad utility in design, optimization and application of new biomaterials, including the development of thermosensitive biogels; but also in the study of thermal regulation in biological materials and tissues in vitro (including also food materials [39, 40]) and in vivo (hyperthermia, cryotherapy and others). The theoretical concept as well as the algorithms used for the calculation of statistical temperature parameters (descriptors) will be presented as a proof of principle. While this theoretical paper is predominantly supported by in-silico data, a comprehensive report currently in preparation will include both extended in-silico and numerous hydrogel ^1^H NMR experiments that validate our concept and provide application examples.

## Background and algorithms

### Conditions for determining temperature heterogeneity by ^1^H NMR spectroscopy

The use of ^1^H NMR spectroscopy for temperature measurement is based on the temperature dependence of the chemical shift, δ_H2O_, of the water resonance. The relationship between δ_H2O_ and the sample temperature, temp, is virtually perfectly linear between room and physiological temperatures, and very close to linear between 0 and 100°C [23, 27]:

$$\text{temp}={\text{a}}_{0}+{\text{a}}_{1}\times \delta_{{\text{H}}_{2}\text{O}}$$ (1)

where a_0_ and a_1_ are empirical values that have to be determined by way of calibration measurements. These values vary slightly as a function of the material under consideration. As an example, for hydrogel loaded with mammalian cells in culture medium under physiological conditions, a_0_ = 471.8°C and a_1_ = -93.4°C/ppm [27]. Moreover, to make appropriate use of eq 1, chemical-shift referencing is required as described in Materials and Methods.

Our method for quantitative characterization of temperature *distributions* presented here is based on the following new, general paradigm: Suppose that a sample is heterogeneous with respect to a measurement parameter, *p*. Further suppose that the chemical shift of an NMR resonance is a function of *p*. Then, the statistical distribution of the *p* values within the sample can be obtained by appropriate line shape analysis of said NMR resonance.

To derive a temperature distribution from a water ^1^H NMR resonance, the NMR resonance needs to be converted to a temperature curve using eq 1 above. This temperature profile then represents the temperature distribution within the measured volume. Note that the resulting curve may also be influenced by other contributions to the line shape: (i) by magnetic-field inhomogeneity and transverse relaxation (T_2_) processes; (ii) potentially by uneven free-water distribution across regions of varying temperature; and (iii) by the spectral processing parameters chosen, in particular filter parameters. In heterogeneous materials, the T_2_ effect (natural line width) is often much smaller than the T_2_* effect (line width dominated by magnetic-field inhomogeneity). Therefore, it is suitable to focus on optimizing the magnetic field homogeneity to minimize T_2_* effects on line width, and to use adequate, constant filter parameters to compare statistical descriptors of temperature heterogeneity between measured samples. In the special case of applications to biological tissue, in particular in vivo, T_2_* effects are due to microscopic variations in magnetic susceptibility. This applies to all temperature measurements by tissue water ^1^H NMR, and is not specific to our new method. Such effects may principally arise from blood since soft tissue is perfused by blood, and the magnetic susceptibility of blood depends on the oxygenation status of red blood cells. The oxygenation status, in turn, determines the relative concentrations of Fe^2+^ vs. Fe^3+^ in hemoglobin. Also other paramagnetic ions such as Mn^2+^ can be at the origin of T_2_* effects. Furthermore, if significantly different tissue types are comprised within a voxel used for temperature measurement (e.g., soft brain tissue along with cerebrospinal fluid), the water protons of that voxel will be characterized by multiple T_2_ values. Therefore, voxels should be chosen judiciously, even though the overall line shape of water protons is clearly dominated by T_2_* rather than T_2_ effects. Of course, in the presence of very strong susceptibility gradients (caused by, e.g., air pockets, strong paramagnetic centers, or even ferromagnetic materials), line shape distortions will be too strong to be dealt with; such NMR spectra will contain susceptibility artifacts and should not be evaluated.

Even with all precautions taken as recommended above, line shape contributions attributable to factors other than temperature-dependent δ_H2O_ may influence experimentally determined temperature curves for very small temperature gradients covering only a few °C. The consequences of contributions from factors other than temperature effects on water ^1^H chemical shift are, of course, reduced precision of our temperature curves and, consequently, reduced precision of the statistical parameters extracted from these curves. The limitations imposed by these imprecisions are material-dependent and have been validated in great detail in a separate report, as pointed out above in Introduction. They become significant when the temperature range covered by the temperature distribution curve is of the same order as, or smaller than, the uncertainty introduced by the spurious effects. However, our new deconvolution procedure is able to largely compensate for these spurious effects on temperature curves in most cases (see S2 File).

In summary, a temperature curve obtained with eq 1 accurately depicts the underlying temperature heterogeneity, within the limits described above. Such a curve is, in fact, an envelope representing the sum of all thermal environments existing within the measured volume. Thus, all thermal environments combined cover the range of temperature values given by the resulting temperature curve. However, to this date no effort has been made to characterize and interpret such envelope curves, judging by published literature. In the following we propose to analyze temperature curves by multiple statistical methods, to extract a number of parameters providing quantitative information on the nature of thermal heterogeneity.

### Parameterizing thermal heterogeneity by statistical descriptors

The most basic objective of statistical analysis of temperature in heterogeneous aqueous materials is the determination of a temperature value that is representative of the entire temperature distribution. Simply using the highest point of the overall curve, as commonly practiced, may be misleading in the case of an asymmetric temperature distribution because this choice would overrepresent sample regions with temperatures close to the maximum value, and neglect regions represented by an extended flank (tail) of the temperature distribution. We suggest a new strategy providing accurate, weighted-average temperature values ($\bar{\text{temp}}$), and several additional statistical parameters describing thermal heterogeneity. This strategy is based on the concept that the temperature curve calculated from a water ^1^H NMR spectrum can be approximated as a histogram. Such a histogram is formed by using the intensities of the digital points of a temperature curve as heights of the corresponding histogram bars (details are described in the following paragraph). The abscissa values of the histogram are identical with the temperature values of the digital points of the temperature curve, as pointed out above (eq 1). All algorithms used for calculating thermal heterogeneity parameters correspond to established statistical algorithms; however, the original equations have been adjusted for temperature curve-derived histograms rather than conventional histograms.

**Fig 1:**
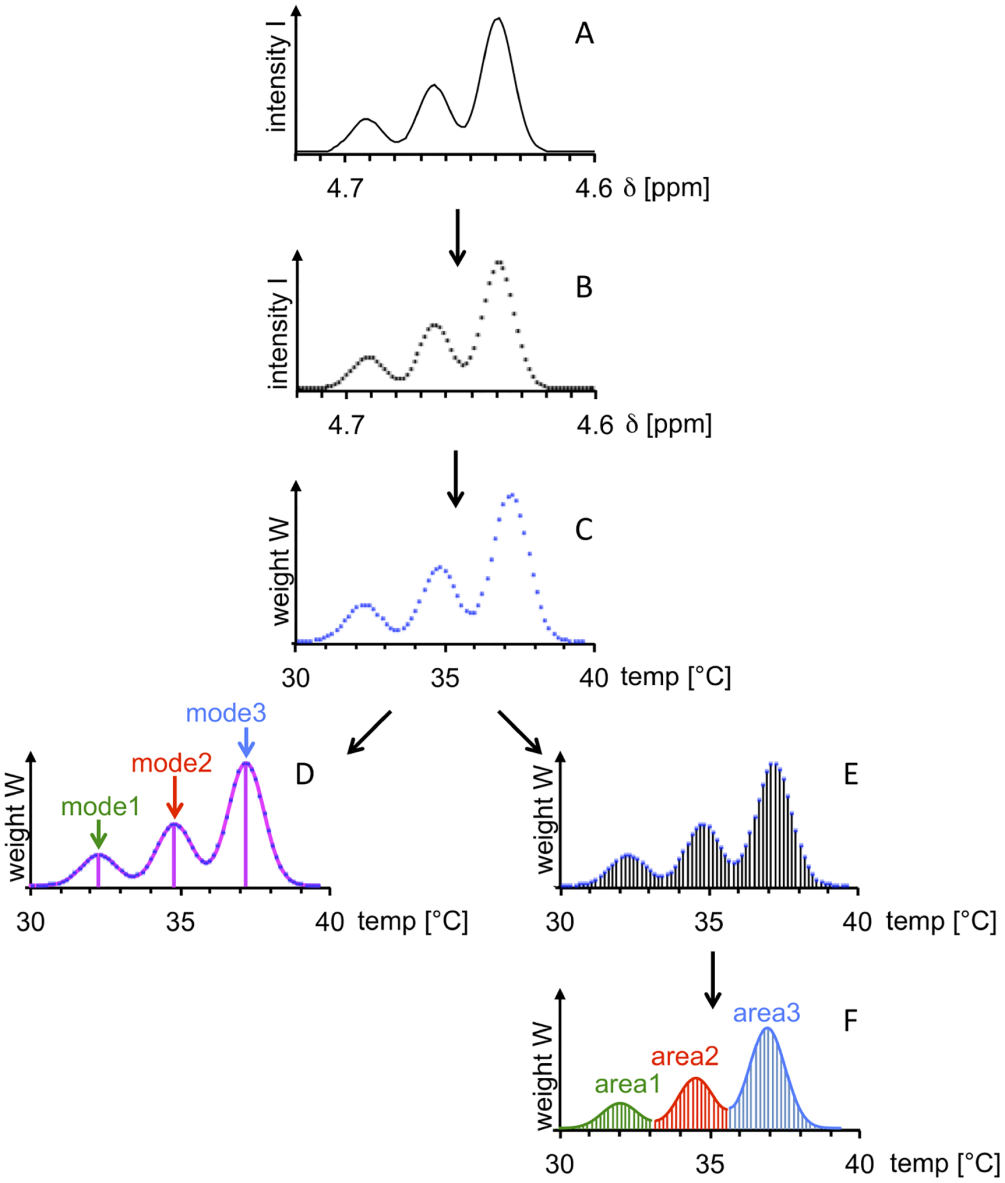
Conversion of a trimodal water ^1^H NMR resonance into a temperature distribution curve. Positions and intensities (weights) of digital points are indicated by points in (B) to (E), and by vertical lines in (E). A) Simulated water ^1^H NMR spectral region before ppm-to-temperature conversion. B) Water spectral region as in (A), represented by the evenly spaced digital points of the spectrum without fitted curve. The height of each digital point is given by I (intensity), its position on the spectral axis by δ (chemical shift). C) Data points as in (B), after ppm-to-temperature conversion. The pattern defined by the digital points is unchanged compared to (B) due to the linear relationship between the chemical-shift (ppm) and temperature (°C) scales. The resulting weight W corresponds to the spectral point intensity I shown in (B). D) Temperature profile generated by curve fitting to the data points represented in (C). In this example, three temperature maxima (modes) can be easily identified. E) Histogram: temperature distribution represented by vertical bars generated by connecting the digital points from (C) with the abscissa. The length of each bar corresponds to its weight W. F) Temperature distribution as shown in (E). The envelope of the temperature distribution is identical to the temperature curve shown in (D). The area under the curve is subdivided into individual color-coded regions associated with the modes identified in (D). This schematic figure exemplifies the following procedures: (i) point-by-point conversion of chemical shift to temperature values, (ii) subsequent generation of an (unbinned) temperature histogram based on digital points, and (iii) visualization of the resulting temperature curve, modes, and individual regions (sub-areas under a curve) associated with these modes. In experimentally obtained spectra, lineshapes are always influenced by factors unrelated to temperature distribution (significant for temperature distributions over small temperature ranges); these are dealt with prior to chemical-shift-to-temperature conversion.

The most important steps in the calculation of statistical temperature heterogeneity descriptors based on a water ^1^H NMR signal are shown in Fig 1 (schematic simulated spectrum). Note that this figure exclusively serves to exemplify the principles of (i) converting the chemical-shift values of digital NMR spectrum points into temperature values, and (ii) evaluating, on this basis, the statistical properties of the resulting temperature distribution curve. If a temperature curve as "pure" as possible is to be generated from an experimental water NMR resonance, spurious contributions, discussed in the preceding subsection, have to be minimized (in experimental setup, spectral processing) and/or compensated for (by spectral post-processing) in a separate procedure prior to chemical-shift-to-temperature conversion. In an NMR spectrum, the intensity I represents the height of a digital curve point as a function of the resonance frequency (here: chemical-shift scale δ in ppm; Fig 1A). The abscissa values for the equidistant digital points of a water ^1^H NMR spectral line (Fig 1B) are directly converted from δ to temp values according to eq 1 (Fig 1C). Connecting the points results in a temperature distribution curve that may show one or several temperature maxima (modes) (Fig 1D). The temperature distributions given in Fig 1C and 1D can also be represented by a histogram (Fig 1E). Wherever meaningful, the total area under the temperature distribution curve can be subdivided, and the resulting sub-histograms can be evaluated individually (Fig 1F, color-coded subregions). For instance, the areas under the subregions of the temperature distribution curve can be quantified to calculate area ratios, area1: area2: area3. All four diagrams (Fig 1C to 1F) represent the same temperature distribution. The shapes of these distributions are identical to the shapes of the underlying spectral line (Fig 1A and 1B), which is a consequence of the strictly linear relationship between δ and temperature.

### Temperature curves as histograms

**Fig 2:**
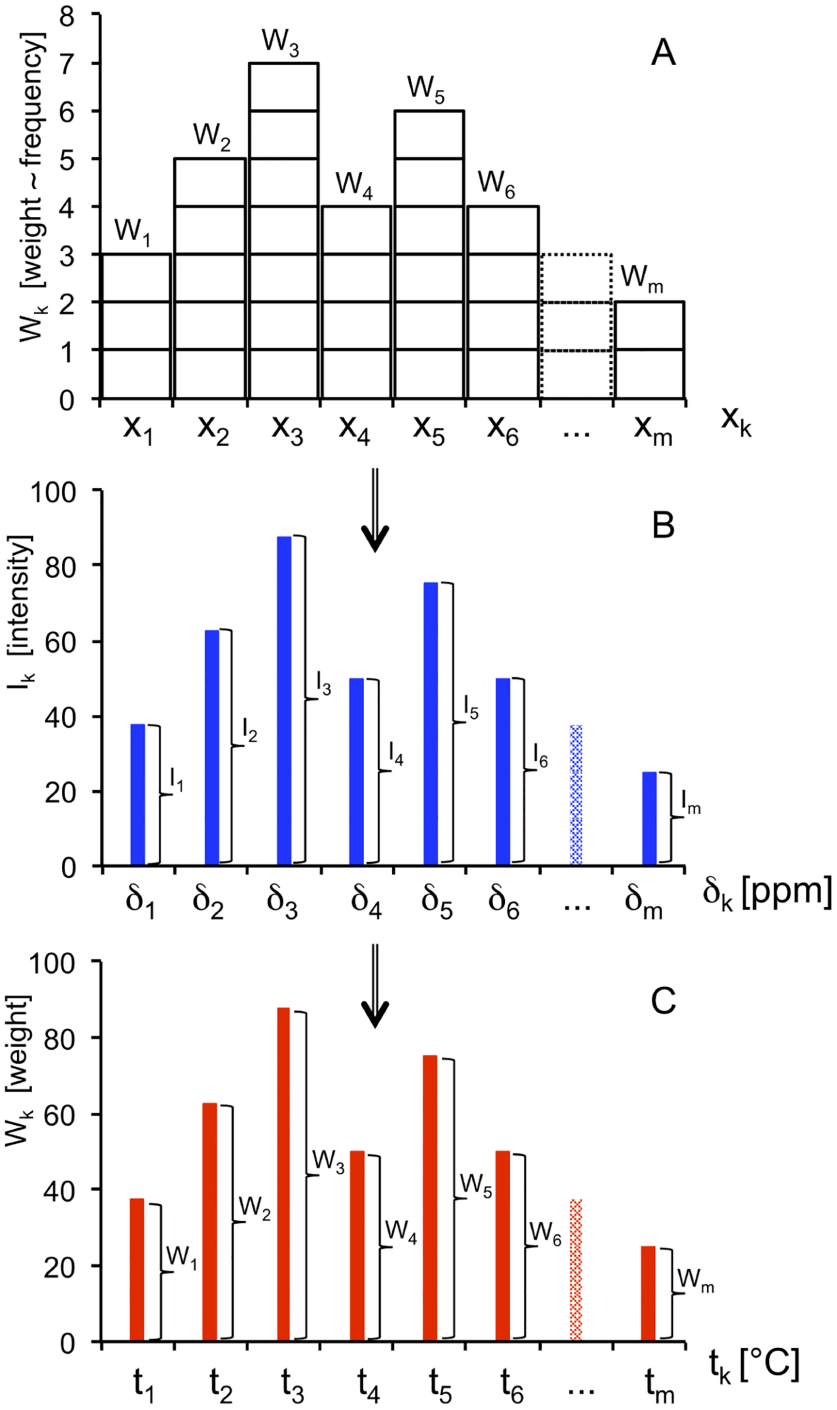
Graphical presentation of (A) a conventional histogram based on bins (buckets), and analogous histograms based digital points of (B) an NMR spectral line, and (C) a temperature distribution curve derived from (B). A) The number (frequency) of individual observations associated with each bin x_k_ (from k = 1 to k = m) corresponds to weight W_k_ (in this example: W_1_ = 3; W_2_ = 5; W_3_ = 7; W_4_ = 4; W_5_ = 6; W_3_ = 4;…; W_m_ = 2). Each rectangle represents an individual observation (= individual contribution to the distribution function). The total number of observations n equals the sum of all weights:$\text{n}=\sum_{\text{k}=1}^{\text{m}}{\text{W}}_{\text{k}}$. B) Intensity values I_k_ (arbitrary unit) of the digital points δ_k_ (from k = 1 to k = m) of an NMR spectral line (in this example: I_1_: I_2_: I_3_: I_4_: I_5_: I_6_:…: I_m_ = 3: 5: 7: 4: 6: 4:…: 2, by analogy to (A)). The sum of all intensity values is $\sum_{\text{k}=1}^{\text{m}}{\text{I}}_{\text{k}}$. Each vertical bar has been placed at the center of each bin of (A). C) Weights W_k_ (arbitrary unit) of digital temperature curve points t_k_ (from k = 1 to k = m) derived from NMR spectral point intensities I_k_ shown in (B). W_1_: W_2_: W_3_: W_4_: W_5_: W_6_:…: W_m_ = 3: 5: 7: 4: 6: 4…: 2, by analogy to (A) and (B). The sum of all weights is equivalent to the nominal sum n of all (hypothetical) contributions to the entire distribution:$\text{n}=\sum_{\text{k}=1}^{\text{m}}{\text{W}}_{\text{k}}$. This schematic figure exemplifies the relationship between conventional histograms based on binned data (A), and our histograms based on unbinned, discrete data (B, C). For spurious effects on experimental spectra, see legend to Fig 1.

A classical histogram is a representation of a discrete probability distribution, and is built from a frequency table based on a total of n independently measured parameter values [41]. Each observation (measured value), indicated by an individual rectangle in the schematic Fig 2A, belongs to one of m adjacent categories x_k_, from k = 1 to k = m. The category axis becomes the abscissa of the histogram.

The weight W_k_ of each category x_k_ is proportional to the frequency of observations (measurements) falling into this category. Thus, W_k_ necessarily is an integer. Weights are indicated by the heights (or areas) of vertical columns (sums of identical rectangles, Fig 2A). Consequently, the total number of measurements n equals the sum of all weights:$\text{n}=\sum_{\text{k}=1}^{\text{m}}{\text{W}}_{\text{k}}$. If the observed parameter is a continuous variable x, the total range of this variable is broken down into a number of equal intervals x_k_ ('buckets' or 'bins') from k = 1 to k = m. Taken together, the frequencies with which a measured parameter falls into all of these intervals x_k_ constitute the frequency distribution.

Modern NMR spectrometers acquire and process spectra as digitized data sets. In practical terms, an NMR spectrum consists of a sequence of equidistant digital points that can be thought of as representing a statistical frequency distribution (ordinate) of chemical shifts δ (abscissa); however, the m intervals (bins) of classical histograms (Fig 2A) are substituted with m discrete values of the measured variable δ_k_ from k = 1 to k = m (Fig 2B). Moreover, the heights of the digital points of a spectrum do not reflect sums of individual measurements, but represent signal intensities at curve points, I_k_. For this reason, the weights derived from NMR spectra are rational numbers rather than integers. After δ (ppm) values are converted point by point to temperature (°C) values, the abscissa is made up of discrete temperature values, t_k_ (Fig 2C). In analogy to Fig 1, spurious lineshape contributions are not discussed for Fig 2 or in the ensuing derivation of our algorithms, and are dealt with separately.

### Weighted mean and median temperature

The conversion of digital temperature curves to histograms as introduced in the previous paragraph, permits the calculation of weighted-average temperature values and other statistical parameters in analogy to established algorithms. In these histograms, the weight, W_k_, of any given bin is defined as the intensity of the corresponding digital point in the temperature curve: W_k_ = I_k_, where k is the index counting the histogram bins or digital curve points to be used for calculating the weighted average from k = 1 to k = m. Based on these curve points, a weighted-average temperature value can be readily obtained by multiplying the temperature value, t_k_, of each digital point by its weight, W_k_, and by dividing the sum of these products by the sum of all weights, as described in S2 File. Besides $\bar{\text{temp}}$ calculation, I_k_ is used in the calculation of the weighted median $\tilde{\text{temp}}$, but also in the determination of (i) skewness, (ii) kurtosis and (iii) entropy of temperature distributions as well as (iv) distinct areas under temperature curves (see below). Note that due to the linearity between δ and temperature, sequential ppm-to-temperature conversion of the equidistant digital points of a spectrum results in equidistant points on the temperature scale. Major advantages of using a weighted-average temperature value compared with the temperature of a single (i.e. the highest) curve point are: (i) $\bar{\text{temp}}$provides an unbiased mean temperature that truly represents the entire temperature range, (ii) $\bar{\text{temp}}$can be obtained regardless of the shape of the temperature distribution (broad or narrow; symmetric or asymmetric; unimodal, bimodal or multimodal), and (iii) ^1^H NMR lineshape distortions caused by factors other than temperature have little influence on the resulting $\bar{\text{temp}}$ value since lineshapes are affected by these factors independently of temperature, unless magnetic-field inhomogeneity is extremely large *and* varies significantly between volume regions characterized by different temperatures. In many applications it may be possible, and even preferable, to reference the water chemical shift to a resonance whose chemical shift is independent of temperature.

Akin to $\bar{\text{temp}}$, median temperature, $\tilde{\text{temp}}$, provides an unbiased temperature value that represents the entire temperature range; it can be obtained regardless of the shape of the temperature distribution; and lineshape distortions caused by other factors than temperature have little influence on the resulting $\tilde{\text{temp}}$ value. In addition, a well-known advantage of medians vs. means is that the former are more robust to outliers. Weighted-median temperature was determined according to an algorithm that is essentially equivalent to the general algorithm for median calculation from a frequency distribution [34, 42, 43].

### Temperature skewness, kurtosis and entropy

Histograms constructed as described above can be analyzed for skewness and kurtosis in temperature. Since skewness is a measure of the lack of symmetry of a given distribution [34], temperature skewness = G1_temp_ = 0 for perfectly symmetric normal temperature distributions, whereas G1_temp_ < 0 (> 0) for temperature distribution curves with a relatively heavy left (right) tail. Since kurtosis determines to what degree a distribution is peaked or flat relative to a normal distribution, kurtosis of a normal temperature distribution = G2_temp_ = 0 [34, 44]. Our equations for skewness and kurtosis calculation were adopted from the statistics module of the EXCEL spreadsheet, and adapted to temperature distributions as described in S2 File. While kurtosis can be used as a measure of the peakedness or flatness of a heterogeneous temperature distribution, also the evenness (smoothness) of a temperature curve can be determined by employing a statistical function known as standard entropy, H_S_ (discrete Shannon entropy [45–47]). Temperature entropy, based on the equation given in S2 File, is a direct measure of how even a temperature distribution is: low entropy indicates that there are significant sample volumes with particular temperature values that occur at much higher frequencies than other regions within the measured volume [42].

### Temperature modes, ranges and volume regions

**Fig 3:**
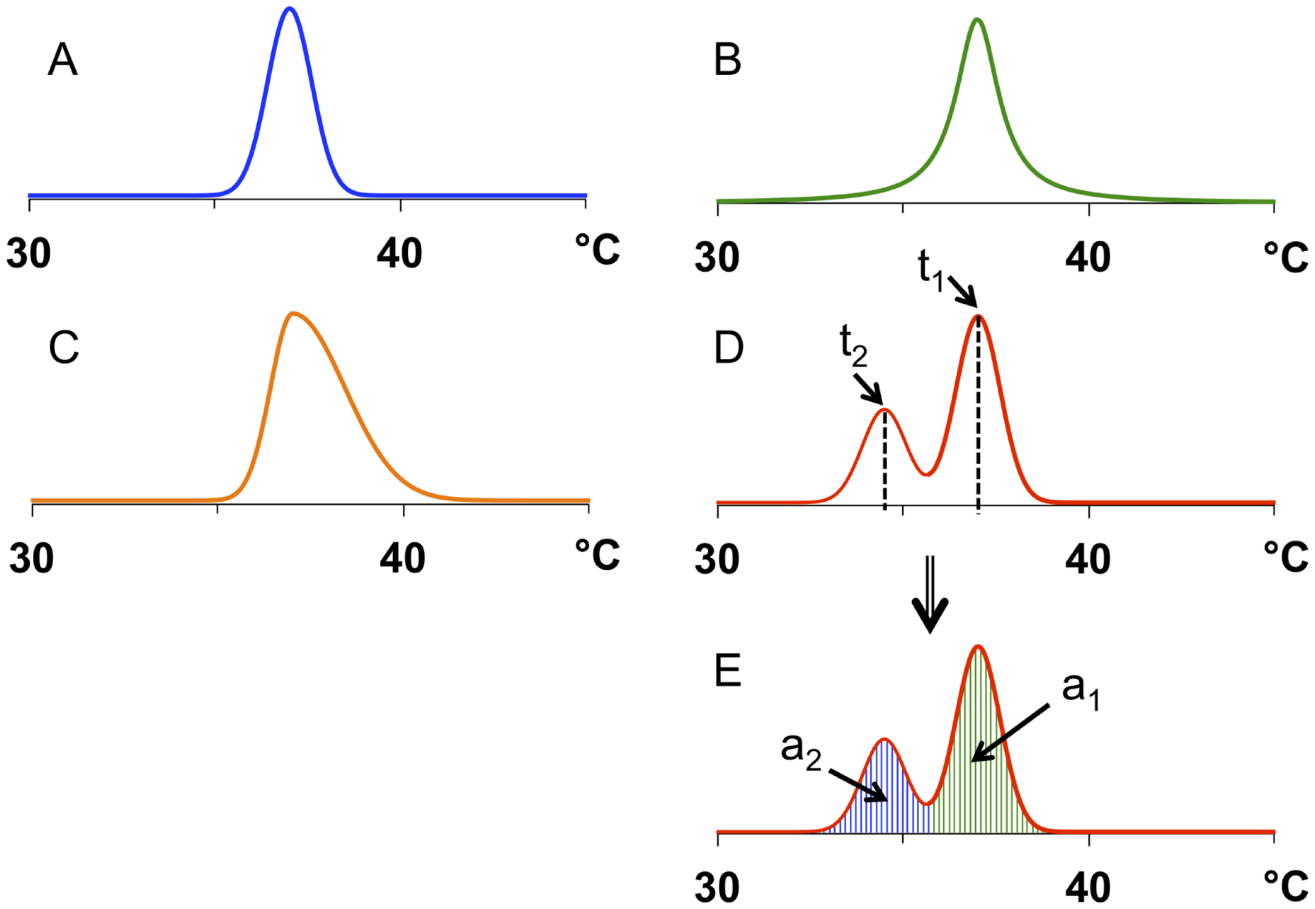
Basic simulated ^1^H NMR-derived line shapes used for in-silico modeling of temperature distributions. The corresponding values for statistical descriptors are given in Table 1. A) Gaussian distribution curve centered about 37°C. The full width at half maximum (FWHM) was chosen to be on the order of line widths (7.5 Hz) obtainable in gel or tissue water ^1^H NMR spectra at 500 MHz under ideal experimental conditions and for minimal temperature variance:$\text{FWHM}=2\sqrt{2\text{ln}2}\times \sigma$. Here, the Gaussian standard deviation, σ, corresponds to s, the nominal standard deviation of our algorithm. B) Lorentzian temperature distribution centered about 37°C, with a similar line width as for (A). C) 'Asymmetric Gaussian' temperature distribution 'centered' about 37°C. For temperature values < 37°C, the same s value as in (A) was chosen. For temperature values > 37°C, an s value twice as large as in (A) was chosen. D) Bimodal temperature distribution based on two superimposed Gaussians centered about 34.5 and 37°C, with s values as in (A). t_1_ and t_2_: temperature modes 1 and 2. E) Bimodal temperature distribution as in (D), with characteristic color-coded sub-regions of the area under the distribution curve. a_1_ and a_2_: areas associated with modes 1 and 2.

In statistics, the mode is the value that occurs most frequently in a data set or a probability distribution, and a multimodal distribution is a continuous probability distribution with two or more modes [34, 42, 48]. The distribution of temperature values across a given volume of heterogeneous material may be multimodal, as described above. This type of temperature distribution manifests itself by two or more maxima (= modes) in a temperature distribution curve. Their associated temperatures (positions of the corresponding **maxima**, Figs 1D and 3D) can often be determined individually, along with the corresponding peak heights. Likewise, multiple characteristic temperature **ranges** (Figs 1F and 3E) in a temperature curve can be frequently identified by determination of their left and right limits; the corresponding individual delimited areas can be quantified by integration as described below. In summary, the temperature modes and temperature ranges-based methods presented here have the advantage of yielding more detailed temperature information than the commonly used practice of determining one overall maximum only for an entire temperature curve. Our approach amounts to an identification of multiple "subpopulations" of sample subregions, where each subpopulation is characterized by a specific temperature distribution centered about a distinct, dominating temperature value, and/or by a specific temperature range. Further details concerning the background of and algorithms for our statistical descriptors of temperature distributions are provided in S2 File.

## Results and discussion

### Application of temperature heterogeneity algorithms to Gaussian and Lorentzian line shapes

The ability of our approach to provide the statistical descriptors of temperature distribution mentioned above is best demonstrated by applying our algorithms to several well-defined computer-simulated line shapes. Gaussian curves possess rather light tails; therefore, extreme points do not contribute significantly to temperature distribution curves (Fig 3A, 3C to 3E). However, for better precision the data point range considered should comprise extended tails where these are present, particularly for line shapes with markedly Lorentzian character (Fig 3B). In Gaussian distributions, skewness, G1, and kurtosis, G2, are zero by definition. Using our algorithm, G1 = G2 = 0.000 was obtained for a set temperature range of at least 5.898°C, and a line width of 7.5 Hz for simulated Gaussian temperature distributions (Fig 3A and Table 1, column A). Narrowing the range resulted in less precise, i.e., small finite G1 and G2 values. Note that the nominal standard deviation, s, corresponds to the well-defined Gaussian standard deviation, σ, resulting in a Gaussian line width, $\text{FWHM}=2\sqrt{2\text{ln}2}\times \sigma$(FWHM = full width at half maximum). However, σ is not well-defined for distributions other than Gaussian. Although Lorentzian distributions are perfectly symmetric, we obtained a G1 value slightly smaller than 0.000 (Fig 3B and Table 1, column B), despite the choice of a very large temperature range (60°C) almost perfectly centered about 37°C. Obviously, the extremely extended tails of a Lorentzian render skewness sensitive even to temperature curve points very distant from the center of the distribution.

**Table 1:** Statistical descriptors characterizing temperature heterogeneity, based on simulated and experimental temperature distribution curves.

descriptor	A^[a]^	B^[b]^	C^[c]^	D/E^[d]^	H^[g]^	H^[h]^
weighted mean, $\bar{\text{temp}}$[°C]	37.02	37.00	37.66	36.18	41.42	41.10
weighted median,$\tilde{\text{temp}}$ [°C]	37.02	37.00	37.54	36.62	48.17	48.44
mode, t_1_ [°C]	36.95	36.95	37.09	36.95	15.30	14.44
mode, t_2_ [°C]	-	-	-	34.43	53.41	54.55
standard deviation, s [°C]	0.59^[f]^	3.62^[f]^	1.03^[f]^	1.33^[f]^	14.99	14.78
peak area ratios (a_2_/a_1_) ^[e]^					a_1_/a_2_ ^[i]^	a_1_/a_2_ ^[i]^
- fitted	-	-	-	0.50:1		
- integrated	-	-	-	0.49:1	0.49	0.85
peak height ratios (h_2_/h_1_) ^[e]^					h_1_/h_2_	h_1_/h_3_
- fitted	-	-	-	0.50:1		
- at curve max.	-	-	-	0.50:1	0.38	0.46
skewness, G1	0.000	-0.013	0.588	-0.506	-0.732	-0.736
kurtosis, G2	0.000	20.779	0.256	-0.959	-0.945	-1.034
standard entropy, H_S_	4.133	5.775	4.880	4.990	8.168	7.841
range, r [°C]	5.88^[f]^	59.96^[f]^	9.25^[f]^	8.13^[f]^	66.56	46.64

A to D/E: values derived from the corresponding simulated curve shapes presented in Fig 3A to 3E.

^[a]^ Gaussian distribution.

^[b]^ Lorentzian distribution.

^[c]^ asymmetric Gaussian distribution.

^[d]^ bimodal distribution based on two overlapping Gaussian distributions.

^[e]^ a_n_ and h_n_: areas and peak heights, respectively, associated with modes t_n_, for n = 1 or 2 (or 3 for experimental curve).

^[f]^ s and r: judiciously chosen values; note that by definition, both are infinitely large for ideal Gaussians and Lorentzians.

^[g]^ and [h]: values obtained without [g] or with [h] correction by reference deconvolution of the underlying hydrogel water ^1^H NMR spectrum.

^[i]^ a_1_/a_2_: for these particular experimental curves, comparison of area ratios is of limited value since deconvolution resulted in significant change of curve shape.

The very high G2 value was to be expected in view of the narrow tip and extended tails of Lorentzians, as was the increased nominal standard deviation that proved to be higher than that of any other simulated distribution shown in Fig 3 and Table 1. Note that for measured curves, the temperature range usable for descriptor calculations would be smaller than for simulated curves because it is limited by background noise; thus, G2 values as low as 0.000 are highly unlikely even if the discernible line shape should be perfectly Gaussian.

For perfectly symmetric distributions such as Gaussian and Lorentzian (Fig 3A and 3B; Table 1, columns A and B), mode, weighted mean and weighted median should yield identical values. In practice, mode may deviate from $\bar{\text{temp}}$ and $\tilde{\text{temp}}$ by a minute amount since its precision depends on the distance between the digital points near the curve maximum. Note that the temperature mode values given in Table 1 refer to discrete curve points rather than interpolated curve maxima. This choice is in keeping with the classical statistical definition of modes corresponding to discrete histogram bins. However, if mode were to be determined by interpolation, e.g., by defining the position of the maximum as a variable to be fitted to an appropriate, mathematically defined curve shape, the resulting value could be considered to represent the "real" maximum of the distribution curve. This method is known as peak fitting in NMR spectroscopy, although it may prove to be difficult to identify appropriate fit functions for many temperature distributions. By contrast, the use of digital curve points for temperature mode definition as suggested in this work is generally applicable, and would result in ambiguity only if two adjacent curve points at the curve maximum have identical heights, which is however highly unlikely.

For positively and negatively skewed temperature distributions (Fig 3C, 3D and 3E, respectively), G1 showed positive and negative values as expected (Table 1, columns C and D/E, respectively), regardless of their monomodal (C) or bimodal (D/E) nature. The ranges chosen were almost identical for these distributions. Note that in practice, the useful range of a temperature distribution may be limited by the signal-to-noise ratio of the underlying NMR spectrum. However, for our (noise-free) simulated distribution curves, maximal range values were determined beyond which there were no significant effects on range-sensitive statistical descriptors, notably kurtosis. Since the bimodal distribution chosen has a dip rather than a peak at the center, its G2 value was negative, as opposed to the moderately positive G2 value found for the asymmetric monomodal temperature distribution with one marked peak in addition to a heavy tail. Entropy values were not dramatically different between the temperature distributions presented, but were, unsurprisingly, relatively high for the distribution with the heaviest tail (Lorentzian; Fig 3B and Table 1, column B), and relatively low for the distribution with the lightest tail (single Gaussian; Fig 3A and Table 1, column A). The theoretical peak height and area ratios for the bimodal temperature distribution (Fig 3D and 3E) are a_2_/a_1_ = h_2_/h_1_ = 0.5, based on the parameters used for numerical simulation of the two Gaussians. Since the overlap of the two curves is only moderate, we obtained ratios identical to or very close to the ideal values (Table 1, column D/E). Thus, the theoretical soundness of our paradigm for quantitative statistical characterization of thermal heterogeneity by water ^1^H NMR spectroscopy has been demonstrated for all statistical descriptors suggested.

### Example of an application of temperature heterogeneity algorithms to a measured water ^1^H NMR line shape: The hydrogel experiment

**Fig 4:**
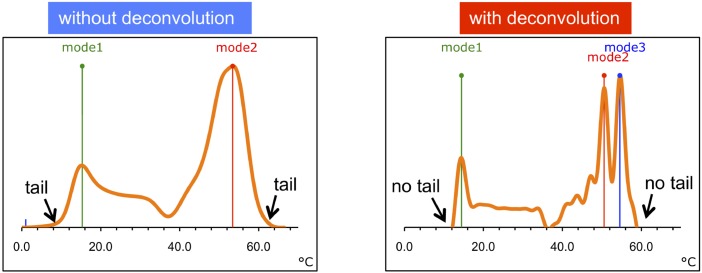
Temperature distribution curve measured by ^1^H NMR spectroscopy. The underlying hydrogel spectrum was obtained based on the coaxial-tube experiment described in the text. Left: Temperature distribution based on the spectrum without deconvolution correction. Right: Temperature distribution based on the same spectrum, after deconvolution with a water resonance obtained at thermal equilibrium. [Deconvolution performed with the EXCEL calculation template provided in S1 File.] The corresponding temperature distribution descriptor values are presented in columns H of Table 1.

An experimental proof of principle for our new method was obtained through a water ^1^H NMR spectrum from a dedicated hydrogel sample exposed to strong temperature gradients. This sample was generated by inserting a cold hydrogel-filled NMR tube into a wider coaxial tube filled with hot hydrogel, as described in Methods. After spectrum processing and chemical shift-to-temperature conversion, the two peaks of an essentially bimodal temperature distribution can be distinguished (Fig 4, left).

The right (high-temperature) peak is larger than the left (low-temperature) peak, in agreement with the outer volume being about three times as large as the inner volume in the combined sample. Some limited heat exchange between the two compartments is reflected by the finite curve intensity between the two peaks indicating a temperature difference of about 40°C at the time of measurement (see also Table 1, last two columns). Temperature gradients of this order of magnitude may be relevant in studies aimed at testing the behavior of aqueous materials. When chemical shift-to-temperature conversion was performed after deconvolution of the underlying spectrum with a reference spectrum obtained on the same sample in the absence of temperature gradients, a temperature curve with better resolved details was obtained (Fig 4, right). Comparison of the two curves demonstrates that line shape contributions imposed by factors other than temperature (T2, T2*) may obscure some details of a given temperature distribution, although overall shapes of the two curves are rather similar. For most statistical temperature heterogeneity descriptors shown in Table 1 (last two columns), there is no dramatic difference between curves with and without deconvolution. However, the values for temperature ranges were substantially altered by reference deconvolution. This is due to the fact that the outer tails of the underlying NMR resonance approach the baseline more or less asymptotically, due to the inherent T2* and T2 effects on any NMR line shape. NMR line shapes are also influenced by the filter parameters chosen (usually Lorentzian, Gaussian, or a combination of both). In summary, our deconvolution method serves to (i) enhance the resolution of the resulting temperature curve and thus provide more details of the actual temperature distribution, and (ii) remove "artificial" tails from the overall temperature curve, thus providing a realistic value for the true underlying temperature range. Further comprehensive validation will be provided in a forthcoming study based on both simulated and experimental temperature distributions.

## Conclusion

We have presented here a method for quantifying thermal heterogeneity in aqueous materials. Our approach is based on a new paradigm suggesting statistical line shape analysis of water ^1^H NMR signals which are considered as approximations of temperature histograms. The most important statistical descriptors of temperature distribution that can be derived from our analysis have been presented. Further descriptors can be adopted from classical distribution statistics; it remains to be seen which descriptors will turn out to be most useful ones in practical applications. A comprehensive validation of our concept and suggested algorithms, comprising analyses of a broad range of line shapes using in-silico and in-vitro NMR experiments, will be presented separately, in conjunction with applications to dynamic changes of temperature gradients.

## Materials and methods

### In-silico techniques and spectrum processing

All computer simulations and calculations were performed using EXCEL spreadsheets programmed with the algorithms presented in this work (EXCEL for Macintosh vs. 14.4.7, Microsoft, Redmond, WA, USA). A detailed manual describing the use of this spreadsheet is provided as a document embedded in the EXCEL file temp_param_template.xlsx (S1 File). Briefly, the mathematical function for δ-to-temperature conversion (eq 1) was used to transform the ppm units of the digital points of an experimental spectrum to °C units. The underlying spectrum may be the uncorrected raw spectrum, or the raw spectrum corrected by our deconvolution procedure using an experimental reference spectrum. The resulting data were plotted to generate a temperature profile (= temperature distribution curve). Interactive selection of the relevant spectral region was employed to determine the range of the temperature curve. This range was used to calculate the statistical descriptors (mean, median, mode(s), skewness, kurtosis, entropy, nominal standard deviation, integrated areas under the curve (see BACKGROUND AND ALGORITHMS section). However, fitted areas under the curve were determined by using the appropriate procedure (mdcon = "mixed deconvolution" command) of our spectrum processing software (TopSpin 1.3, Bruker, Rheinstetten, Germany), after importing the simulated temperature curve. For more technical details concerning in-silico line shape simulation and processing, see S2 File.

### Water ^1^H NMR spectroscopy of a dedicated hydrogel sample

A test sample designed to provide genuine temperature gradients was generated as follows. An alkaline (pH 8.2) gel sample containing 1% agarose and 20 mM phosphorylcholine as chemical-shift reference was filled into a 5-mm NMR tube which was then put on ice. In addition, an alkaline gel sample containing 1% agarose and 20 mM *N*-acetylaspartate as a chemical-shift reference was filled into a 10-mm NMR tube, which was then maintained at 60°C in a water bath. Subsequently, the 10-mm tube was removed from the water bath, the cold 5-mm NMR tube was inserted into the hot 10-mm tube, and a ^1^H NMR spectrum was immediately acquired from the combined sample in the 10-mm NMR probe of an AVANCE 400 WB spectrometer (Bruker, Rheinstetten, Germany). The ^1^H NMR acquisition at 400 MHz was based on a simple one-pulse sequence with one transient (NS = 1) and a very small flip angle (1.5°), a signal (FID) acquisition time of 0.41 s corresponding to 4 k data points, and a sweep width of 12.4723 ppm. The acquisition of our spectrum was timed so as to occur before substantial heat exchange could take place between the tubes, with the aim of demonstrating the presence of rather large temperature gradients.

## Supporting information

S1 File Supplemental software. Example and template for calculation of statistical temperature distribution descriptors.
(XLSX)

S2 File Supplemental theory. Further details on paradigms and algorithms.
(PDF)
