# Supplementary material for: Multiparametric quantification of thermal heterogeneity within aqueous materials by water 1H NMR spectroscopy: Paradigms and algorithms
Source: PLoS One. 2017 May 26;12(5):e0178431. doi: 10.1371/journal.pone.0178431 (PMC5446178; doi:10.1371/journal.pone.0178431)
Supplement: S2 File — Further details on paradigms and algorithms. (PDF) [file pone.0178431.s002.pdf]

S-2 File. Supplemental Theory

Multiparametric Quantification of Thermal Heterogeneity Within Aqueous Materials by Water ^{1}H NMR spectroscopy: Paradigms and Algorithms

Norbert W. Lutz, Monique Bernard

Aix-Marseille Université, Faculté de Médecine, CNRS UMR 7339, Centre de Résonance Magnétique Biologique et Médicale, F-13385 Marseille Cedex 5, France

Use 'bookmarks' for quick overview of, and direct access to items contained in this document

SUPPLEMENTARY THEORY

Table of Contents

Page

1. In-silico line shape simulation S-2
2. Further algorithms and equations for statistical descriptors of thermal heterogeneity S-2
   2.1. Temperature curves as histograms; weighted mean and median temperature S-2
   2.2. Temperature skewness, kurtosis and entropy S-6
   2.3. Temperature modes, ranges and volume regions S-8
3. Temperature curve processing S-9
4. Correction of temperature curves S-9
   4.1. Compensation of spurious line shape modifications by using the convolution theorem S-9
   4.2. Compensation of the temperature dependence of water spectral line widths S-12
5. Thermal macroheterogeneity vs. microheterogeneity S-12
   5.1. Principle of intravoxel vs. intervoxel thermal heterogeneity S-12
   5.2. Comparison of spectrum-based vs. image-based thermal heterogeneity analysis S-17
6. References S-18

1. In-silico line shape simulation. EXCEL spreadsheets were programmed to simulate basic line shapes. For Gaussian lines, the following equation was used:

$$I_K = h \times e^{-\frac{(\delta_k - \delta_T)^2}{2\sigma^2}} \qquad \text{S-1}$$

where I_k = intensity of digital point k of the water ^{1}H NMR resonance, from k = 1 to k = m; m = total number of digital points making up the used spectral line; h = height of the curve's peak; δ_k = chemical shift of digital point k of the water ^{1}H NMR resonance, from k = 1 to k = m; δ_T = chemical shift of the water ^{1}H NMR resonance corresponding to temperature T at the center of the Gaussian line; σ = standard deviation controlling the width of the Gaussian line, with $\sigma = \frac{LW}{2 \times \sqrt{(2\ln 2)}}$ and LW = line width [ppm] corresponding to 7.5 Hz.

For "asymmetric" Gaussians (Fig. 3 C), LW = 7.5 Hz for $(\delta_k - \delta_T) > 0$, and LW = 15 Hz for $(\delta_k - \delta_T) < 0$. Chemical shifts δ_T were 4.6918, 4.6648 and 4.6378 ppm for 32.0, 34.5 and 37.0 °C, respectively. To simulate full ^{1}H NMR spectra with 8 k data points, δ_k was varied in 8192 equidistant steps between 10.995 and –1.003 ppm (intervals of 0.0014648 ppm). For h, relatively high values were chosen to ensure that n >> m, as explained elsewhere [1]: h = 10000 for the maximum at 37 °C (unimodal, bimodal and trimodal distributions), and h = 2500 and 5000 for maxima at 32.0 and 34.5 °C, respectively, for bimodal and trimodal distributions. The latter were constructed by superimposing Gaussians with the corresponding temperature maxima and h values (Figs. 1 A - F, and 3 D,E).

For Lorentzian lines, the following equation was used:

$$I_k = \frac{h}{(\frac{\delta_k - \delta_T}{LW/2})^2 + 1} \qquad \text{eq. S-2}$$

with parameter values as given above for Gaussians (unimodal distribution only).

2. Further algorithms and equations for statistical descriptors of thermal heterogeneity.

2.1. Temperature curves as histograms; weighted mean and median temperature. In an NMR spectrum presented as a histogram, each curve point is characterized by an abscissa value, t_k, and a weight, W_k, for k = 1 to k = m (Fig. 2 C). The histogram then reflects the relative prevalence of t_k values in the measured sample. As indicated in the main body of this paper, the weight W = W_k of a particular digital point k in a temperature curve or histogram

reflects the relative frequency with which the temperature value represented by this point occurs in the measured sample (Fig. 2 C). W_k can be thought of as the sum of (real or hypothetical) individual contributions to a given digital point k of the temperature curve. It follows that $n = \sum_{k=1}^{m} W_k$ is the sum of the weights of all digital points of the temperature curve [39]. By analogy to conventional histograms, n can be considered as the (real or hypothetical) total number of individual contributions to the temperature frequency distribution, and interpreted as being proportional to the total number of equal microscopic sample volume elements contributing to the weights W_k of the digital points k of the temperature curve. The following equation was used for determining weighted mean temperature:

$$\overline{\mathrm{temp}} = \frac{\sum_{k=1}^{m}(t_k \times W_k)}{\sum_{k=1}^{m} W_k} \qquad \text{eq. S-3}$$

where W_k is the weight of digital point k of the temperature curve, t_k is the discrete temperature value of digital point k, and m is the number of digital point k of the temperature curve.

As outlined in the main body, water ^{1}H NMR lineshape distortions caused by factors other than temperature have little influence on the resulting $\overline{\mathrm{temp}}$. In this context, the question of possible line shape effects may be raised for reference signals as well. A number of small organic molecules may serve as internal references, depending on the application in question. For biological tissues, such compounds may be phosphorylcholine (PCho) or *N*-acetylaspartate (NAA), both of which are characterized by methyl singlet positions invariant to temperature. Obviously, for extremely inhomogeneous magnetic fields the lineshapes of such reference signals would be distorted along with the water signal, which would complicate temperature analysis. In this case, alternative correction methods should be considered, as presented below. For less distorting magnetic-field inhomogeneities, it is sufficient to calibrate the weighted-average chemical shift of the reference signal to its set value prior to ppm-to-temperature conversion.

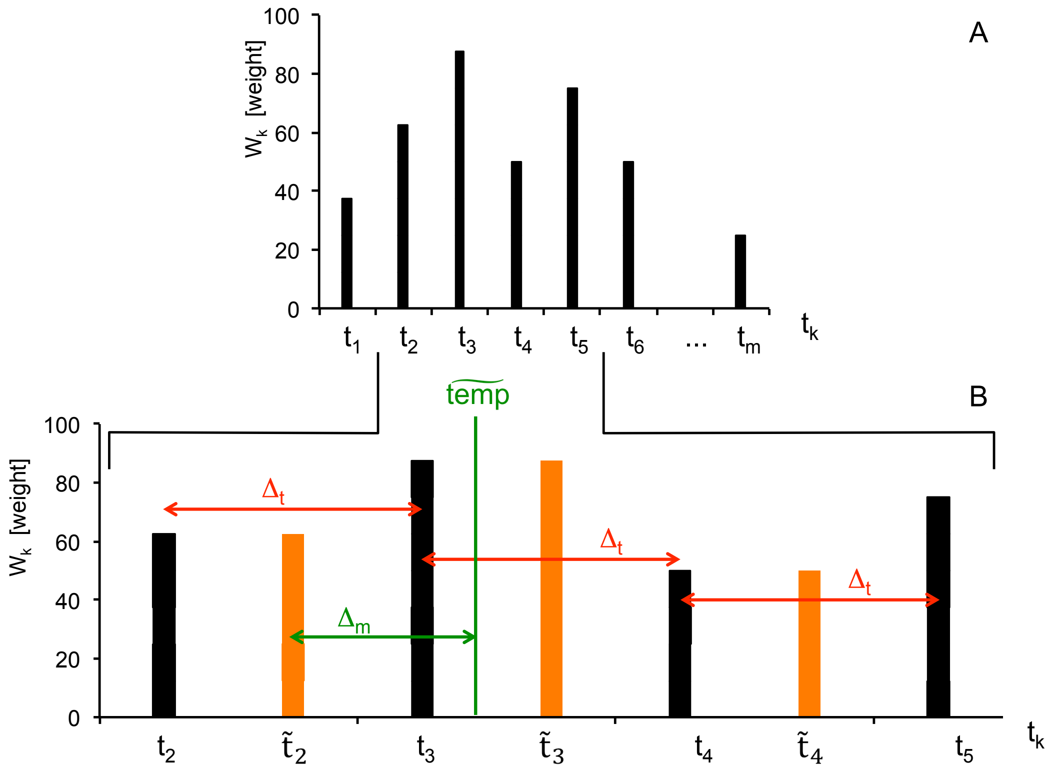

Figure S-1 : Determination of the weighted median temperature, $\widetilde{temp}$, of a temperature distribution.

(A) Weights W_k for temperature distribution curve points t_k, from t_1 to t_m. The first step in $\widetilde{temp}$ calculation is the determination of cumulated sums CSUM (Eqs. S-4 and S-5), with $\mathrm{CSUM(m)} = \sum_{k=1}^{m} W_k$.

(B) Section of histogram shown in (A), with additional parameters needed for $\widetilde{temp}$ calculation. Δ_t, interval between two adjacent points of the temperature distribution curve. Δ_t values are constant across the entire temperature distribution due to the linear relationship between δ and temperature. Black bars: weights of temperatures, as defined in (A). Orange bars: weights of temperatures corrected for discrete vs. binned-value histogram ($\tilde{t}_k = t_k + \frac{\Delta_t}{2}$). Δ_m, interval between $\widetilde{temp}$ (green vertical line) and the next lower temperature after $\frac{\Delta_t}{2}$ correction (in this example, $\tilde{t}_2$).

Since the median is generally more robust to outliers and extreme values than the mean, small foci of atypical temperature values in materials with otherwise "typical" temperature distributions will influence the median temperature less than the median temperature. Therefore, depending on the purpose of temperature analysis, either $\overline{temp}$ or $\widetilde{temp}$ should be emphasized. Weighted median temperature was determined via calculation of cumulative sums CSUM (corresponding to cumulative frequencies in histograms) for the weights of the individual temperature curve points: $\mathrm{CSUM(k)} = \sum_{i=1}^{k} W_i$, for all k from k = 1 to k = m. Then, the cumulative sum of weights

obtained for the last point of the temperature curve, $\mathrm{CSUM(m)} = \sum_{i=1}^{m} W_i$, is divided by 2, yielding the half-sum $\mathrm{CSUM(h)} = \frac{\mathrm{CSUM(m)}}{2}$. Subsequently, the temperature curve point k whose cumulative sum equals the obtained half-sum is identified, i.e., the curve point k for which $\mathrm{CSUM(k)} = \mathrm{CSUM(h)}$. The temperature value of curve point $k = h$, i.e., $t_k = t_h$, is defined as the weighted median temperature, $\widetilde{temp}$.

Although this relationship may be directly applied to t_k defined in the main part of this report, two adjustments are needed to obtain correct temperature values for h and, subsequently, accurate $\widetilde{temp}$ values[1]. First, the algorithm described above has originally been established for frequency distributions based on binned measurement of continuous parameters, as outlined in this report. In this context, the cumulative sum of each bin k includes all observations from parameter interval 1 through parameter interval k. By contrast, our temperature histograms are not based on continuous-parameter measurements that are assigned to bins because each point k represents a discrete temperature value. In fact, a t_k value can be defined as a distinct data point (Fig. 2 C) located at the center of a conventional bin interval x_k (Fig. 2 A). For this reason, the temperature values t_k used for the identification of $\widetilde{temp}$ as described in the previous paragraph (Figs. S-1 A and B, black bars), need to be corrected by adding to each t_k one half of $\Delta_t = (t_{k+1} - t_k)$, i.e., the distance to the next curve point, such that $\tilde{t}_k = t_k + \frac{\Delta_t}{2} = t_k + \frac{1}{2}(t_{k+1} - t_k)$ (Fig. S-1 B, orange bars). Second, in most real-world cases none of the points $\tilde{k}$ represented by a $\tilde{t}_k$ value possesses a cumulative sum $\mathrm{CSUM}(\tilde{k})$ that is exactly equal to the half-sum, $\mathrm{CSUM(h)}$. Instead, there will be two adjacent points, $\tilde{k} = \tilde{k}_h$ and $(\tilde{k} - 1) = (\tilde{k}_h - 1)$, that possess cumulative sums such that $\mathrm{CSUM}(\tilde{k}_h - 1) < \mathrm{CSUM(h)} < \mathrm{CSUM}(\tilde{k}_h)$. In other words, the exact median temperature will lie between these adjacent temperature curve points and can be interpolated based on the differences between the half-sum $\mathrm{CSUM(h)}$ on the one hand, and each of the cumulative sums, $\mathrm{CSUM}(\tilde{k}_h - 1)$ and $\mathrm{CSUM}(\tilde{k}_h)$, on the other. Consequently, the equation to be used for the calculation of the weighted median temperature is

$$\widetilde{temp} = \tilde{t}_{kh-1} + \Delta_m = \tilde{t}_{kh-1} + (\tilde{t}_{kh} - \tilde{t}_{kh-1}) \times f_{int}\ , \qquad \text{eq. S-4}$$

where $\tilde{t}_{kh}$ is the temperature value of curve point $\tilde{k}_h$ possessing the cumulative sum $\mathrm{CSUM}(\tilde{k}_h)$; $\tilde{t}_{kh-1}$ is the temperature value of curve point $(\tilde{k}_h - 1)$ possessing the cumulative sum $\mathrm{CSUM}(\tilde{k}_h - 1)$; and f_{int} is an interpolation factor defined as

f_{int} = [CSUM(h) – CSUM($\tilde{k}_h$−1)] / [CSUM($\tilde{k}_h$) – CSUM($\tilde{k}_h$−1)] eq. S-5

It is evident that $\widetilde{temp} = \tilde{t}_{kh}$ in the limit where CSUM($\tilde{k}_h$) = CSUM(h). Likewise, $\widetilde{temp} = \tilde{t}_{kh-1}$ in the limit where CSUM($\tilde{k}_h$−1)= CSUM(h).

2.2. Temperature skewness, kurtosis and entropy. Our equations for skewness and kurtosis calculation were adopted from the statistics module of the EXCEL spreadsheet and adapted to temperature distributions:

$$\text{temperature skewness} = \mathrm{G1}_{\mathrm{temp}} = \frac{n}{(n-1)(n-2)}\sum_{l=1}^{n}\left(\frac{t_l-\overline{temp}}{s}\right)^3 \quad \text{eq. S-6}$$

$$= \frac{n}{(n-1)(n-2)}\sum_{k=1}^{m} W_k\left(\frac{t_k-\overline{temp}}{s}\right)^3$$

$$\text{temperature kurtosis} = \mathrm{G2}_{\mathrm{temp}} = \frac{n(n+1)}{(n-1)(n-2)(n-3)}\sum_{l=1}^{n}\left(\frac{t_l-\overline{temp}}{s}\right)^4 - \frac{3(n-1)^2}{(n-2)(n-3)} \quad \text{eq. S-7}$$

$$= \frac{n(n+1)}{(n-1)(n-2)(n-3)}\sum_{k=1}^{m} W_k\left(\frac{t_k-\overline{temp}}{s}\right)^4 - \frac{3(n-1)^2}{(n-2)(n-3)}$$

with $$s = \sqrt{\frac{\sum_{l=1}^{n}(t_l-\overline{temp})^2}{(n-1)}} \quad \text{eq. S-8}$$

$$= \sqrt{\frac{\sum_{k=1}^{m} W_k(t_k-\overline{temp})^2}{(n-1)}}$$

and $$n = \sum_{k=1}^{m} W_k, \quad \text{eq. S-9}$$

where s is the nominal standard deviation, a parameter analogous to the standard deviation of the mean based on individual observations; t_l is the temperature value of the l-th (real or hypothetical) individual contribution to the measured temperature distribution; l is a parameter analogous to the index that counts individual observations in conventional skewness and

kurtosis calculation, from l = 1 to l = n. As pointed out elsewhere in more detail, skewness and kurtosis become independent of n for n greater than several times the number of digital points m [1]. Therefore, to obtain consistent results it is sufficient to scale the weights W_k such that n is much greater than m (W_k is typically on the order of several thousand or higher).

The interpretation of temperature profiles in terms of skewness and kurtosis is straightforward, based on the definitions presented above and in the main body. Skewness > 0 indicates a higher prevalence of high-temperature vs. low-temperature sample volumes. This implies that the low-temperature (< mean temperature) sample volume fraction consists of regions whose temperature values vary little and are close to the mean, while there is a relatively large temperature variability within the high-temperature sample volume fraction. The opposite applies to distributions with skewness < 0. For kurtosis > 0, a considerable proportion of the sample volume has temperature values that fall within a very narrow range around the center of the temperature distribution, while the residual sample volume has temperature values that are spread over a large range beyond the center (tail-heavy temperature distribution). By contrast, for kurtosis < 0 the bulk of the sample volume has temperature values distributed over a significant range around the mean (flat temperature distribution), but very little sample volume has extreme temperature values (shoulder-heavy temperature distribution). Accordingly, both skewness and kurtosis are parameters useful for characterization of temperature heterogeneity.

When the concept of entropy is applied to the analysis of the weights W_k of a temperature distribution,

$$\text{temperature entropy} = H_{temp}(W) = H_S = -\sum_{k=1}^{m}\left[\frac{W_k}{\sum_{k=1}^{m} W_k}\log_b\left(\frac{W_k}{\sum_{k=1}^{m} W_k}\right)\right], \qquad \text{eq. S-10}$$

where b = 2 is used by convention. Equation S-10 is based on the classical definition of standard entropy, H_s, as described in the main part of this work. However, the standard entropy of a particular distribution of parameter values depends on the number, m, of parameter values. Therefore, if entropies of distributions with significantly different numbers of parameter values are to be compared, it is meaningful to normalize each entropy value with respect to the number of underlying parameter values, according to the following equation:

$$\mathrm{H_n} = \mathrm{H_S}/\log_2(\mathrm{k}) \ , \qquad \text{eq. S-11}$$

where H_n is the normalized entropy.

2.3. Temperature modes, ranges and volume regions. In the presence of major temperature differences, more than two or three separate modes can potentially be detected. In the opposite case, *i.e.,* when the temperature difference between two volume regions is on the order of 2.5 °C or smaller, it will be difficult to clearly separate modes in temperature curves derived from standard water ^{1}H NMR spectra. Instead, temperature "maxima" may appear as shoulders on broad lines, but can still be used to characterize temperature heterogeneity. Relatively flat temperature distributions that do not possess well-defined modes may be frequently found in practical situations, but cannot be mimicked easily in silico or in vitro. However, well-defined unimodal or oligomodal distributions are amenable to illustrating our paradigm and demonstrating the basic proof of principle.

Quantitative characterization of temperature distributions with multiple distinguishable temperature ranges can be achieved by calculating or estimating the overall physical sizes of these regions. Since the temperature curves are based on NMR spectra, the area under a temperature curve is, under appropriate measurement conditions, proportional to the amount of the compound measured. For this reason, we suggest to use ^{1}H NMR-derived temperature curves to quantify overall volume regions defined by specific temperature values. Relative sizes of individual areas under a particular temperature curve can be obtained as a result of (i) fitting of multiple analytical curves in some favorable cases, or (ii) integration over defined temperature ranges. The relative areas (e.g., area ratios) calculated in this way directly yield the corresponding relative volumes, provided that the free-water content does not vary significantly across the regions studied. To obtain accurate area ratios by integration (ii), intensities of curve points are summed over chosen temperature ranges. Fitting of multiple analytical functions with Gaussian/Lorentzian line shapes can be achieved with conventional NMR spectrometer software. Although the latter procedure is frequently referred to as "deconvolution", it should not be confounded with the homonymous mathematical operation also known as "faltung" (folding) which is used by us in the context of "deconvolution" for water signal correction (see below).

3. Temperature curve processing. A detailed description of the steps involved in the statistical analysis of our temperature curves is provided as a document embedded in the EXCEL file temp_param_template.xlsx (Supporting Information). The algorithms used were entirely based on the equations given in the main body of this paper. The first step included the conversion of an NMR spectrum into a temperature distribution curve. This was achieved using the first calculation sheet of our EXCEL file, which exclusively consists of two input columns for spectral data in ASCII format (one column for the chemical-shift values of the abscissa, and one column for the intensity values of the ordinate, i.e., one data pair per digital spectrum point). Relevant spectral and temperature ranges were selected interactively, and were verified by inspecting the automatically updated ranges in the figure windows of this sheet. Relevant temperature ranges were fine-tuned interactively by using the second calculation sheet. This sheet exclusively serves to extract the statistical descriptors of temperature value distributions from the temperature curves. Multiple modes were identified by inspection of the relevant column as described in the manual embedded in the EXCEL spreadsheet. All other statistical descriptors were automatically calculated by the programmed algorithms.

4. Correction of temperature curves. In addition to the temperature-dependence of the chemical shift of water protons, multiple factors may significantly influence line shapes of experimentally determined temperature curves if temperature gradients are very small, i.e., if the temperatures occurring within the measured volume vary only by a few degrees Celsius, or less. Here, we introduce concepts designed to cancel out these spurious effects. The feasibility of our approach is demonstrated below; its full implementation, as well as the evaluation of its potential and limitations are an ongoing effort.

4.1. Compensation of spurious line shape modifications by using the convolution theorem. As outlined in the main body of this manuscript, temperature curves derived from water proton NMR spectra may be significantly influenced by effects of T_2, T_2*, magnetic-field inhomogeneity and other factors. All of these define NMR line shape and line width *in the absence* of temperature gradients. In fact, measured "raw" temperature curves can be thought of as "true" temperature distribution curves (defined solely by the temperature dependence of δ_{H20}) combined with concomitant line shape contributions caused by said spurious effects. It is therefore possible to correct experimentally obtained raw temperature distribution curves for modifications caused by spurious effects. As a first approximation, we propose to use a

reference water NMR resonance from the sample in question, at thermal equilibrium. During the acquisition of this spectrum, the sample temperature should be close to the average temperature of the subsequent measurement(s) to be performed in the presence of temperature gradients. The temperature curve(s) derived from the latter experiment(s) should then be corrected by taking into account the line shape and line width of the reference spectrum obtained at thermal equilibrium.

The separation of two concomitant contributions to NMR line shapes can be obtained by a process known as deconvolution. In physical and mathematical terms, deconvolution of two functions in the spectral (frequency) domain corresponds to a division of the corresponding functions in the FID (time) domain. In the inverse process, convolution of two functions in the time domain corresponds to a multiplication of the corresponding functions in the frequency domain. This is a direct consequence of the well-established convolution theorem [2] stating that the Fourier transform of the convolution of two functions is equal to the product of their individual Fourier transforms,

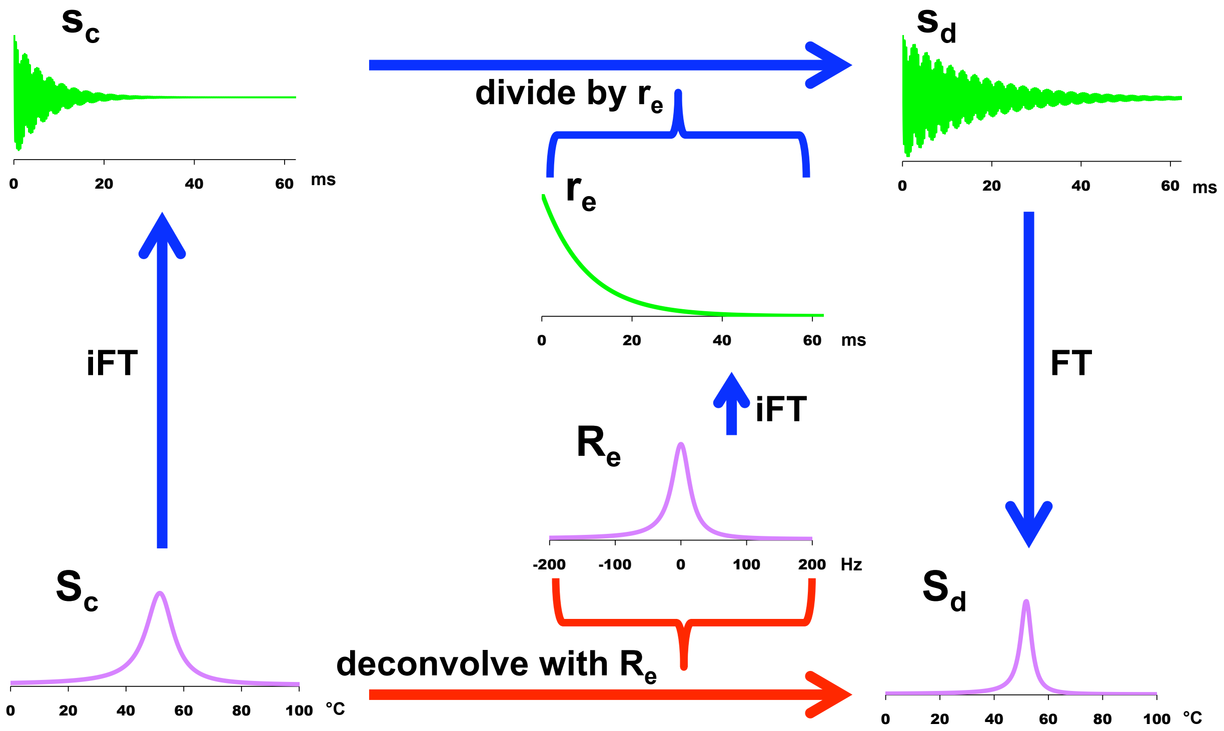

Figure S-2 : The modeled raw temperature distribution curve (bottom left, convolved function) is deconvolved with a function corresponding to a spectral line obtained in the absence of temperature gradients (bottom center, response function) to yield the true temperature distribution curve (bottom right, deconvolved signal function). This transformation procedure is equivalent to, and made practically feasible by (i) converting the convolved and response functions to their corresponding iFTs (top left and center,

respectively); (ii) dividing the iFT of the convolved function by the iFT of the response function (result, top right); and (iii) converting the resulting FID to its corresponding spectral line (bottom right) by FT. In practice, the top left FID is directly available as a result of the NMR experiment, and does not need to be obtained by iFT. Since in this particular *in-silico* example both the convolved and response functions are perfectly Lorentzian, the deconvolved signal is also Lorentzian, as was to be expected based on the convolution theorem. The expected (and desired) consequence of this deconvolution procedure is the reduced line width of the deconvolved signal (bottom right) when compared with the original signal (bottom left). For better visibility, only the initial parts of the FIDs, and the regions around the temperature distribution curves (corresponding to water ^{1}H NMR resonances) are shown.

$$\mathcal{F}\left\{s\otimes r\right\}=\mathcal{F}\left\{s\right\}\times\mathcal{F}\left\{r\right\} \qquad \text{eq. S-12}$$

and, inversely, the Fourier transform of the product of two functions is equal to the convolution of their individual Fourier transforms

$$\mathcal{F}\left\{s\times r\right\}=\mathcal{F}\left\{s\right\}\otimes\mathcal{F}\left\{r\right\} \qquad \text{eq. S-12}$$

where s = signal function, r = response function, and $\mathcal{F}$ = Fourier transform.

Both convolution and deconvolution are widely applied in high-resolution NMR spectroscopy, in particular for spectral filtering (apodization) and resolution enhancement by Lorentzian-Gaussian line shape transformation. We suggest to apply an equivalent approach to the deconvolution of the raw temperature distribution curve (convolved function, $s\otimes r$) with the reference curve (response function, r). This is to be achieved by dividing the FID acquired in the presence of temperature gradients by the inverse Fourier transform (iFT) of the reference spectrum. The Fourier transform of the resulting FID is then easily converted to the true temperature distribution curve (signal function, s) using the algorithms described in the main manuscript. For direct and inverse Fourier transform calculations, EXCEL was employed in conjunction with the StatPlus:mac module, version v5 (AnalystSoft, Walnut, CA, USA).

The suggested deconvolution procedure is demonstrated in Fig. S-2 for a numerically simulated model. In our example, both signal and response functions are Lorentzian. FIDs were modeled by exponentially decaying sinusoids, and the corresponding Lorentzians were obtained by

Fourier transformation. Note that the FID corresponding to the response function has the form of an exponential curve (equivalent to an on-resonance FID).

4.2. Compensation of the temperature dependence of water spectral line widths. The corrections introduced in subsection 4.1. suppose that the response function itself does not vary as a function of the temperature. In particular, the effective line shape and line width underlying the response function were assumed to be constant. These assumptions are rather realistic in cases where the spurious contribution to the measured line shape is determined by magnetic-field inhomogeneity. However, in cases where T_2 processes related to molecular mobility, exchange and/or other factors have a decisive influence on the line width, the line width may significantly change with temperature. For many materials, line widths can be measured as a function of (homogeneous) sample temperature in separate experiments, and the result of these measurements can be included in the mathematical description of the response function. The development of appropriate algorithms for these and further sophisticated corrections will be included in future projects centered about quantitative characterization of temperature heterogeneity by water ^{1}H NMR spectroscopy.

5. Thermal macroheterogeneity vs. microheterogeneity

5.1. Principle of intravoxel vs. intervoxel thermal heterogeneity. In the context of this study, it is important to distinguish between 'temperature regions' (corresponding to temperature ranges) that can be derived from temperature curves, on one hand, and physical regions within a sample on the other. The temperature distributions obtained from ^{1}H NMR spectra approximate histogram-like temperature profiles. Therefore, they do not contain information on the spatial organization of the underlying sample. In particular, no assumptions are made as to the physical shapes, sizes or locations of the physical macro or microregions responsible for the temperature regions, beyond the trivial fact that, e.g., all physical regions measured in one single-voxel ^{1}H NMR experiment are contained within the same macroscopic voxel (voxel = volume element). For instance, the appearance of two distinct temperature regions in a particular temperature profile may be generated by two large volume regions, each one characterized by a distinct characteristic temperature range. However, the same temperature distribution pattern may be produced, in principle, by a mixture of a large number of small volume regions whose temperature values fall into one of the distinct temperature ranges

represented by the temperature profile. In either case, the ratios of volumes characterized by different temperature values can be estimated as described above.

Our technique takes into account thermal heterogeneity independently of its spatial distribution, as opposed to heterogeneities based on temperature maps (such as temperature MR imaging [3-8]). One of these currently existing temperature mapping techniques (MRSI-PRF, magnetic resonance spectroscopic imaging - proton resonance frequency [8]) is based on the same effects of temperature on water proton chemical shifts as our new method presented in this work. To compare the temperature heterogeneities detectable by our proposed method and by temperature maps, it is essential to distinguish between macroscopic thermal heterogeneity (macroheterogeneity) and microscopic temperature heterogeneity (microheterogeneity). The differing consequences of these two forms of heterogeneity on their detectability by image-based and spectroscopy-based temperature measurement techniques are elicited through a gedankenexperiment (thought experiment) presented in Figs. S-3 and S-4. Thermal microheterogeneity refers to temperature differences between physical microregions that are smaller than a voxel underlying a pixel in a temperature map. Thus, several microscopic regions with different temperatures may be located inside the same voxel (microheterogeneity = intravoxel heterogeneity; Fig. S-4, left panels). Microheterogeneity is undetectable in temperature maps because only one pixel value per voxel is obtainable, and this pixel value will

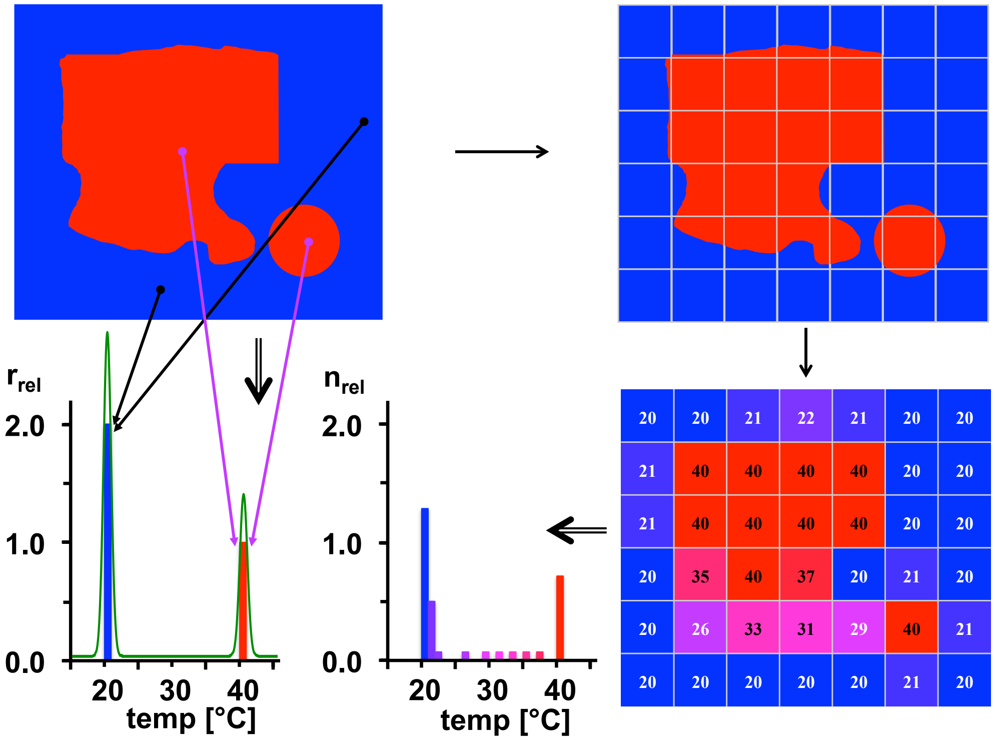

Figure S-3 : Thought experiment for thermal macroheterogeneity

Top left: Cross section of a physical body consisting of macroscopic regions at two different temperatures: 20 °C (blue area) and 40 °C (red areas). The 20 °C area is twice as large as the two 40 °C areas combined.

Bottom left: Histogram accurately representing the temperature distribution for this model. Blue bar and black arrows: 20 °C; red bar and magenta arrows: 40 °C. Relative heights r_{rel} of histogram bars: 2 : 1 (20 °C bar : 40 °C bar). This distribution can be approximated by a temperature curve derived from a water ^{1}H NMR spectrum (green trace; simulated Gaussians).

Top right: Cross section of the same body, with an overlay showing a pixelation grid as used in digital imaging techniques such as MRI. Most pixels are positioned such that they only cover voxels containing either 20 °C or 40 °C regions. However, note that several pixels cover voxels each containing both 20 °C and 40 °C regions.

Bottom right: Digital image generated with the pixelation depicted in the top right panel. "Mixed" pixels that each represent both 20 °C and 40 °C regions, result in averaged (apparent) temperature values that are weighted according to the relative sizes of these regions within each voxel (partial volume effect). The number and hue associated with each voxel represent its apparent temperature [°C]. Despite the partial volume effect, the digital image permits the distinction of the two 40 °C regions from each other and from the 20 °C region, because each of these regions is larger than the pixel size.

Bottom center: Image-based histogram representing the temperature distribution derived from the apparent voxel temperature values for the model (bottom right). The hue of each histogram bar represents

the associated apparent temperature. Note the occurrence of a number of histogram bars at intermediate temperatures; these artifacts are a result of the partial volume effect but do not represent any actual regions within the body volume. The height n_{rel} of each histogram bar is proportional to the number of pixels at the effective temperature in question, and is normalized to the corresponding histogram at the bottom left.

usually represent an average temperature value for the entire voxel (Fig. S-4, right panels). Although intravoxel temperature gradients may be negligible for (i) small thermal gradients throughout the sample and (ii) very small imaging voxels, this would no longer be true for larger thermal gradients within the sample and/or moderate spatial resolution as obtained in many MRSI-PRF experiments.

Thermal macroheterogeneity refers to temperature heterogeneity that is detectable in temperature maps because it is based on temperature differences between (average) temperature values of individual voxels underlying the pixel values in a temperature map (macroheterogeneity = intervoxel heterogeneity; Fig. S-3, right panels). Therefore, temperature imaging detects macroscopic temperature heterogeneity only (histogram in Fig. S-3, bottom center panel). By contrast, our new ^{1}H NMR spectroscopy-based temperature profiles represent macroscopic (histogram in Fig. S-3, bottom left panel) and microscopic (histograms in Fig. S-4, bottom left panels) temperature heterogeneities combined, as these are indistinguishable. The precision of the resulting temperature curves (green traces) may vary as a function of the material being studied; it is only limited by lineshape contributions other than temperature-dependent water proton chemical shift, although much of these spurious effects on linewidths can be minimized and/or compensated for by our deconvolution technique (see section 4 above). In the new statistical temperature profiling method presented in this report, the measured voxel comprises the entire volume of interest, unless multivoxel ^{1}H NMR spectroscopy is performed which produces one spectrum per voxel (briefly discussed in the following subsection). In fact, the issue of macroheterogeneity vs. microheterogeneity is not specific to temperature maps, but is common to all voxel-based imaging [1,9].

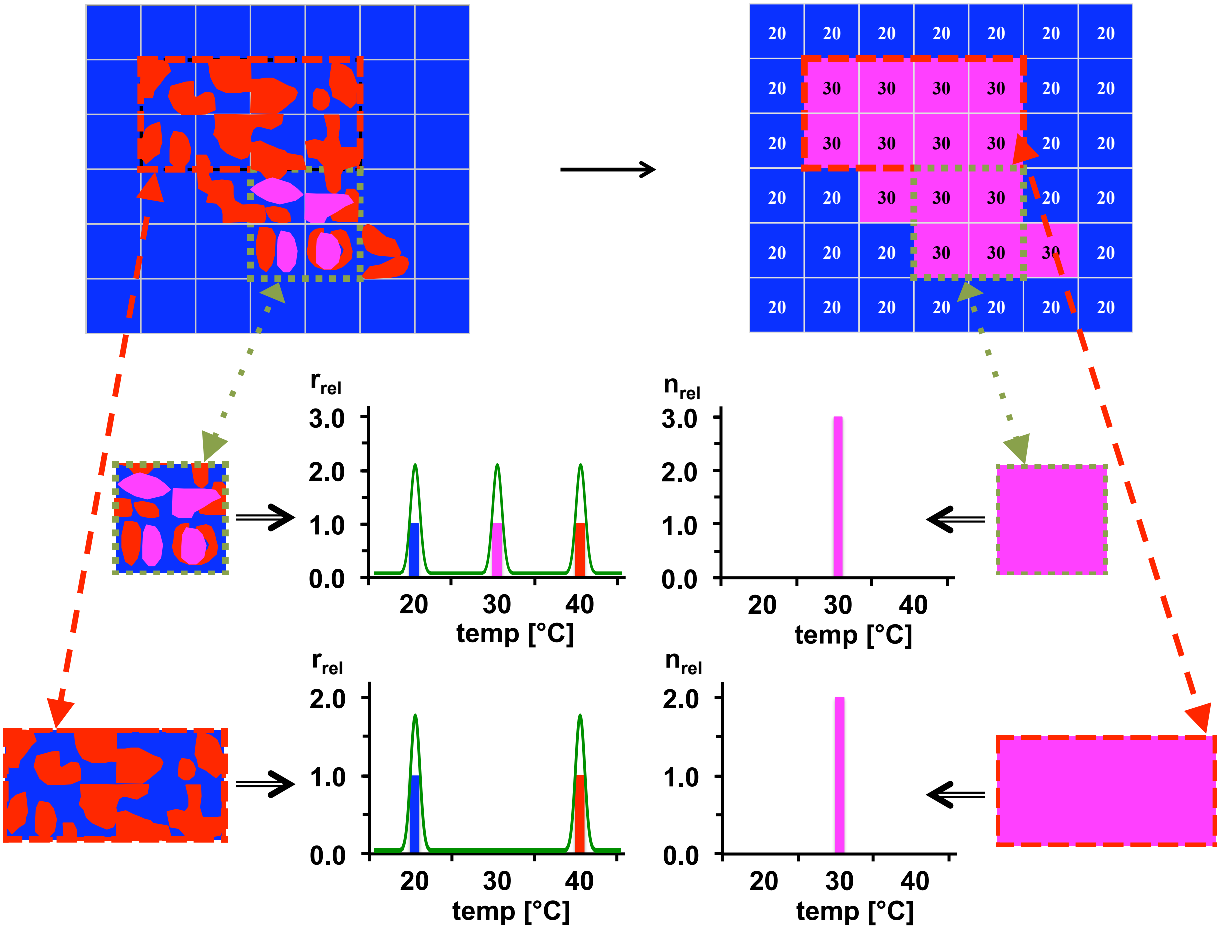

Figure S-4 : Thought experiment for temperature microheterogeneity

Top left: Cross section of a physical body consisting of microscopic and macroscopic regions at three different temperatures: 20, 30 and 40 °C (blue, magenta and red areas, respectively), with a pixelation grid overlay as shown in Fig. S-3, top right. The segment delineated by a green frame only covers pixels each of which represents a voxel consisting of microscopic regions of equal sizes at 20, 30 and 40 °C. The segment delineated by a red frame covers pixels each of which represents a voxel consisting of microscopic regions of equal sizes at 20 and 40 °C.

Bottom left: Histograms accurately representing the temperature distributions for the green and red-framed segments (green and red arrows). The hues of the histogram bars match those of the corresponding microscopic regions. Relative heights r_{rel} of histogram bars: 1 : 1 : 1 (20 °C bar : 30 °C bar : 40 °C bar) for the green-framed segment; and 1 : 1 (20 °C bar : 40 °C bar) for the red-framed segment. These distributions can be approximated by temperature curves derived from water ^{1}H NMR spectra (green traces; simulated Gaussians).

Top right: Digital image generated with the pixelation depicted in the top left panel. "Mixed" pixels each representing equally sized microscopic body regions of 20 °C and 40 °C result in an averaged apparent temperature value of 30 °C (partial volume effect), as do "mixed" pixels each representing equal

microscopic regions of 20, 30 and 40 °C. The number and hue associated with each voxel represent its apparent temperature [°C]. Due to the partial volume effect, the digital image no longer permits the distinction of individual regions based on temperature differences within the heterogeneous core of the body, because these regions are smaller than the pixel size. The only distinction possible is between the periphery (20 °C, genuinely homogeneous temperature) and the core (30 °C, apparently homogeneous temperature).

Bottom right: Image-based histograms representing the temperature for the green and red-framed segments in our model, based on apparent voxel temperature values (see green and red arrows). The hue of each histogram bar represents its apparent temperature, which is identical to that of the corresponding voxels and segments. Note the presence of only one bar in each histogram; this artifact is a result of the partial volume effect in this example of microscopic thermal heterogeneity, and may erroneously suggest thermal homogeneity within the corresponding voxels and segments of the body volume. The height n_{rel} of each histogram bar is normalized to the corresponding histograms at the bottom left.

5.2. Comparison of spectrum-based vs. image-based thermal heterogeneity analysis. In our approach to quantifying thermal heterogeneity, all descriptors are based on digital points of temperature curves derived from water ^{1}H NMR spectra. A crucial aspect of our new paradigm is that these quantitative statistical parameters describe global features of thermal heterogeneity within a selected volume. To the best of our knowledge, none of the currently available temperature measurement methods provides such descriptors. As opposed to voxel-based imaging, our spectroscopic approach indiscriminately takes into account both macroscopic and microscopic heterogeneity, and allows for very fast measurements. Changes in temperature profiles over time can be followed at a rate of several measurements per second, but the temporal resolution is ultimately dependent on physicochemical properties of the aqueous material to be studied. However, the proposed method can be easily combined with one such method that is also based on water ^{1}H NMR (MRSI-PRF). In this way, both statistical and spatial information on temperature distribution can be gained from the same sample, provided the available NMR instrument is equipped for imaging. In fact, our new method could be used to directly analyze water ^{1}H NMR spectra obtained through MRSI-PRF, albeit at the expense of temporal resolution.

6. References

(1) Lutz, N. W.; Le Fur, Y.; Chiche, J.; Pouyssegur, J.; Cozzone, P. J. *Cancer Res* **2013**, *73*, 4616.

(2) Press, W. H.; Teukolsky, S. A.; Vetterling, W. T.; Flannery, B. P. *Numerical recipes in FORTRAN 77. The art of scientific computing*; Cambridge University Press: Cambridge, 1988.

(3) Aime, S.; Botta, M; Fasano, M; Terreno, E; Kinchesh, P; Calabi, L; Paleari, L. *Magn Reson Med* **1996,** *35*, 648.

(4) Coman, D.; Trubel, H.K.; Rycyna, R.E.; Hyder, F. *NMR Biomed* **2009**, *22*, 229.

(5) Liu, G.; Qin, Q.; Chan, K. W.; Li, Y.; Bulte, J. W.; McMahon, M. T.; van Zijl, P. C.; Gilad, A. A. *NMR Biomed* **2014**, *27*, 320.

(6) Marshall, I.; Karaszewski, B.; Wardlaw, J. M.; Cvoro, V.; Wartolowska, K.; Armitage, P. A.; Carpenter, T.; Bastin, M. E.; Farrall, A.; Haga, K. *Magn Reson Imaging* **2006,** *24,* 699.

(7) Thrippleton, M.J.; Parikh, J.; Harris, B. A.; Hammer, S. J.; Semple, S. I.; Andrews, P. J.; Wardlaw, J. M.; Marshall, I. *NMR Biomed* **2014**, *27*, 183.

(8) De Poorter, J. *Magn Reson Med* **1995**, *34,* 359.

(9) Lope-Piedrafita S.; Garcia-Martin, M. L.; Galons, J. P.; Gillies, R. J.; Trouard, T. P. *NMR Biomed* **2008**, *21*, 799.
